# Preliminary results of a new surgical technique: bladder mucosal graft harvested with holmium:YAG (HO:YAG) laser. A new option in bulbar replacement urethroplasty?

**DOI:** 10.1590/S1677-5538.IBJU.2023.9906

**Published:** 2023-05-10

**Authors:** Luiz Augusto Westin, João Boechat, Pedro Gabrich, Felipe Figueiredo, Luciano Alves Favorito

**Affiliations:** 1 UERJ Hospital Universitário Pedro Ernesto Serviço de Urologia Rio de Janeiro RJ Brasil Serviço de Urologia, Hospital Universitário Pedro Ernesto, UERJ Rio de Janeiro, RJ, Brasil; 2 Hospital Pompéia Serviço de Urologia Caxias do Sul RS Brasil Serviço de Urologia, Hospital Pompéia, Caxias do Sul, RS, Brasil; 3 Universidade Estadual do Rio de Janeiro Unidade de Pesquisa Urogenita Rio de Janeiro RJ Brasil Unidade de Pesquisa Urogenital, Universidade Estadual do Rio de Janeiro, UERJ, Rio de Janeiro, RJ, Brasil

**Keywords:** Urethral Stricture, Lasers, Solid-State

## Abstract

**Objective:**

To describe the technique of transurethral harvesting of bladder mucosal graft using the Holmium:YAG (Ho-YAG) laser and describe the preliminary results from 7 cases where this graft was used for urethroplasty.

**Materials and Methods:**

We performed a single-stage dorsal onlay urethroplasty using bladder mucosal graft in 7 patients with anterior urethral stricture. Transurethral harvesting was performed with the Ho-YAG laser. We performed a prospective and descriptive analysis with uroflowmetry performed at 30, 90 and 180 days after surgery and applied the PROM translated into Portuguese before and 6 months after urethroplasty.

**Results:**

Seven patients were included, 2 (28.5%) with penile urethral stricture, and 5 (71.5%) with bulbar urethral stricture. Mean stricture length was 50mm (range 35-60mm). Stricture etiology was trauma in 3 (42.9%) patients, iatrogenic in 1 (14.3%) patient, and idiopathic in 3 (42.9%) patients. Two patients (28.6%) had previously undergone ventral buccal mucosa urethroplasty. Mean bladder mucosal graft length was 52.86mm (± 13.801), and mean harvest time was 46.43min (± 14.639). Dorsal onlay urethroplasty using bladder mucosa was successfully completed in 5 patients (71.4%). Two patients (28.6%) couldn't have the procedure completed using bladder mucosa, one due to thermal damage of the graft during harvesting, and one due to insufficient graft length. In both cases the procedure was completed using buccal mucosa. Two patients (28.6%) experienced minor hematuria between the twelfth and eighteenth postoperative day, but neither required hospitalization and/or additional procedures. All patients achieved normalization of peak flow, and this was maintained throughout the follow-up period. Mean peak flow was 17.8 ml/s (± 3.271) at 30 days, 20.6 ml/s (± 5.413) at 90 days, and 19.6 ml/s (± 8.019) 180 days. Mean IPSS score decreased from 19.3 to 5.4. Similar improvements were also seen in the ICIQ-MLUTS Score (a mean drop from 3.8 to 2.0) and Peeling's Voiding Picture Score (a mean drop from 4.0 to 2.2). Quality of Life improved post urethroplasty, with increases in EQ-5D (from 0.6371 to 0.7285) and EQ-VAS (from 58.0 to 84.0).

**Conclusion:**

Transurethral harvesting of bladder mucosa using the Holmium laser (Ho-YAG) is feasible and reproducible. Our preliminary experience suggests that bladder mucosa grafts achieve comparable results to other grafts when used for dorsal onlay urethroplasty. Further research is needed to confirm these results.

## INTRODUCTION

Urethral stricture disease is defined as a partial or total obliteration of the urethral lumen. In the United States, the estimated prevalence is 0.9% in adult men (1). Most cases are caused by iatrogenic stenoses (43.4%), followed by idiopathic (21.7%), traumatic (21 .5%) and inflammatory causes (13.7%) (2). Urethral Stricture Disease (USD) represents one of the most challenging urological pathologies and is often associated with impaired quality of life (3). Depending upon the patient's age, comorbidities and stage of the disease, patients with USD may be candidates for a series of treatments that vary from urethral dilation to staged urethroplasty, using flaps and grafts.

Replacement urethroplasty involves harvesting of a graft having suitable characteristics and then implanting it into the recipient site for reconstruction of the narrowed urethra. These grafts can be harvested from various sites being either skin or mucosal tissues. Skin graft locations include genital tissues, including preputial skin, penile shaft skin, and scrotal skin. Grafts can also be harvested from mucosal tissues derived from a variety of locations such as: oral mucosa, lingual mucosa, bladder mucosa, and colonic mucosa (3). Oral mucosa and prepuce skin are the tissues being most frequently used.

The ideal graft for urethral reconstruction should be resilient and able to withstand the humid environment, be easily accessible, hairless, and angled allowing for neovascularization (4). Although the use of grafts for urethral reconstruction has been described since the 19th century, it was only popularized by Devine et al. in 1961, when the full thickness of foreskin grafts was used (5). Currently, the type of graft most used for urethral reconstruction is oral mucosa due to the improvement and popularization of the technique developed by Morey and McAninch in 1996 (6).

Bladder mucosa for graft purposes in the treatment of urethral pathologies was first described by Joseph Memmelaar, in 1947 (7). Several authors have since published their experiences using bladder mucosa grafts for urethral stenosis treatment. Many of these publications refer to pediatric and adolescent cases that had severe complications of hypospadias. The surgeries were performed in 1 or 2 stages using multiple techniques for correction of the stenosis (only grafts associated with tubularized grafts), and each performed where the harvesting of the bladder mucosa was done using the open technique (8–10).

In 2018, Wang Z et al. described transurethral harvesting of bladder mucosa graft in 2 patients using the Hybrid Knife system, which uses a jet of water to aid the process. Short-term follow-up did not reveal any bladder complications (11).

The Holmium laser (Ho-YAG) is a pulsed solid-state laser with a wavelength of 2140 nm and penetration depth of 0.4 mm (12). Each pulse is absorbed by water and creates a vapor bubble at the tip of the fiber. When the fiber tip contacts tissue, the laser interacts with the intracellular water and ablate the tissue, creating a cutting effect. When the fiber tip is kept at a distance from the tissue the laser interacts with the irrigation fluid, reducing the amount of energy being delivered to the tissue concurrent (13). A second effect of the laser on tissues is a thermomechanical displacement which is generated by the shock wave of the vapor bubble caused with each pulse. This effect can be exploited by the surgeon to facilitate the enucleation of a prostatic adenoma (14) or in harvesting the bladder mucosa as a free graft.

Our hypotheses were that Ho-YAG could be used for transurethral harvesting of bladder mucosa, and that this graft would achieve comparable results to more commonly used grafts in dorsal onlay urethroplasty. The primary objective of this article is to describe the technique for transurethral harvesting of a bladder mucosal graft using Ho-YAG laser. The secondary objective is to describe the preliminary results of a series of 7 cases using these grafts to perform substitutive urethroplasties.

## MATERIALS AND METHODS

This study received institutional review board approval (IRB number 51456521.8.0000.5259) and was performed in accordance with the hospital's institutional committee's ethical standards for human experimentation.

We prospectively analyzed 7 patients, admitted to our service between November 2021 and January 2023. Inclusion criteria consisted of patients having a diagnosis of anterior urethral stenosis, with or without recurrence, and were indicated for urethroplasty with graft (stenoses greater than 2.5cm). Exclusion criteria included: genitourinary malformations, a history of pelvic radiotherapy, a history of bladder cancer, and those with an indication for staged urethroplasty. Every patient was staged using cystourethrography and uroflowmetry except in those using a suprapubic urinary diversion. The PROMS questionnaire (15) translated into Portuguese was applied preoperatively and then applied again after 6 months of follow-up.

### Equipment needed for graft removal

1. Resectoscope 22 or 18.5F;
2. Working element adapted for laser
3. 550μm laser fiber;
4. High power Ho-YAG laser (minimum 60W)

### Surgical Technique (16)

All surgeries were performed by a single surgeon experienced in urethral surgery. Due to the physical characteristics of the bladder mucosa (soft and tenacious tissue), we chose to perform dorsal onlay (17) or dorsum lateral onlay (18) urethroplasty to avoid diverticula formation.

**Figure 1:**
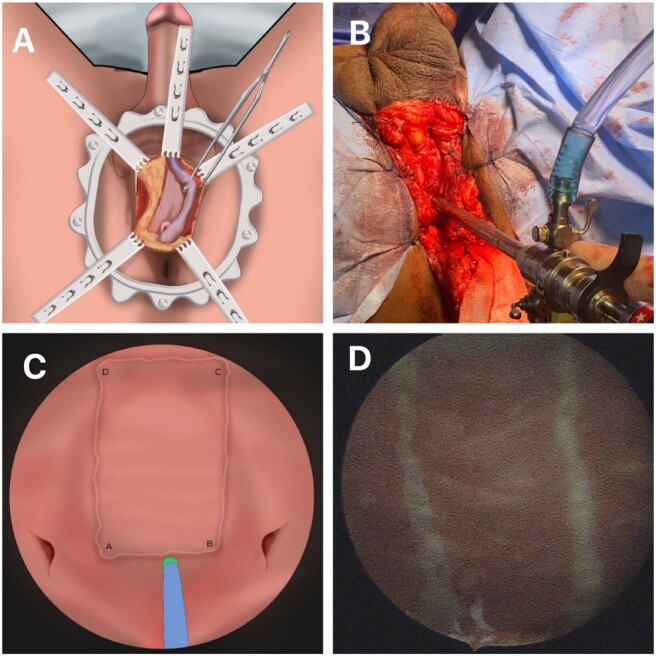
A) Access to the bulbar urethra; Lateral urethral incision with dorsal or lateral dorsum mobilization; stenosis incision; B) Introduction of 18.5 or 22F resectoscope with working element adapted for laser fiber; C) Bladder at maximum repletion. Identification of the ureteral ostia; Rectangular marking of the donor area (apexes A, B, C and D) with 550 μm laser fiber and D) Dissection of the donor area; initially from lateral (CB and/or AD segments) to medial, then from proximal (AB segment) to distal (DC segment).

After placing the patient in the lithotomy position, a perineal incision was made permitting access to the bulbar urethra (Figure-1). The next step proceeded with either the dorsal or lateral dorsum urethral dissection (Figure-1), and then location of the stenosis aided by a urethral catheter, longitudinal section, and measurement of the strictured urethral segment until reaching the suspected healthy proximal and distal urethral areas.

A 22 or 18.5F resectoscope with a working element adapted for the laser fiber was then passed through the proximal urethrostomy (Figure-1) followed by a urethroscopy and cystoscopy using a 0.9% saline solution as irrigation fluid. This is performed to aid in identifying possible bladder and/or urethral pathologies and anatomical landmarks for marking the graft donor region.

**Figure 2:**
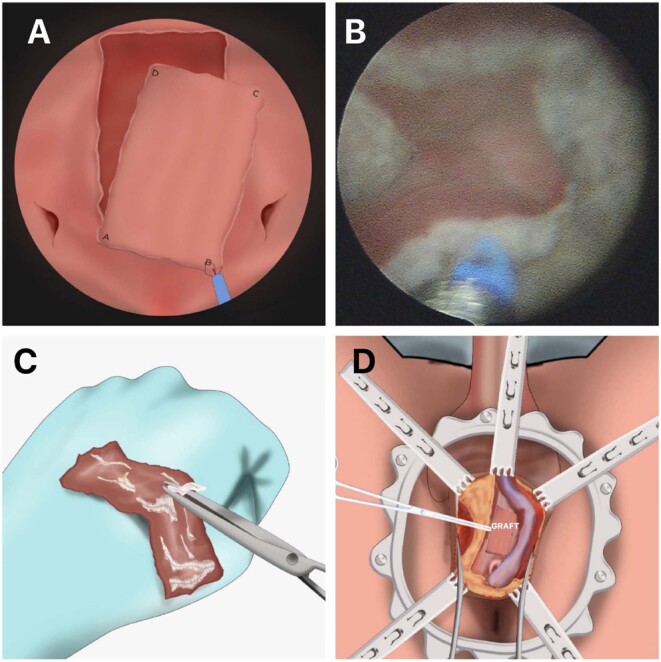
A) Schematic drawing showing the harvesting of the graft from inside the bladder; B) The figure shows the endoscopic view of harvesting of the graft from inside the bladder; C) Preparation of the graft with the removal of fragments of the detrusor muscle adhered to the mucosa and D) Fixation of the graft to the corpora cavernosa with Polydiaxone (PDS) 5-0 and posterior anastomosis between the urethra and the graft with Polydiaxone (PDS) 5-0.

The Holmium Laser settings for energy were 0.5 to 0.8J and frequency of 30 to 40 Hz. After filling the bladder to full capacity, a rectangular marking of the donor graft area was made immediately above the Interureteric bar; its lower and upper sides represented by segments AB and DC, respectively, and its lateral sides represented by segments AD and BC (Figure-2).

Dissection of the graft was then performed using the 550 μm laser fiber, always going from lateral to medial (AD and BC) and subsequently from proximal to distal (AB to DC), being the deepest plane the muscular layer of the bladder. Meanwhile, the bladder was kept fully distended (Figure-2). Upon completing dissection, the graft was extracted from the bladder's interior using forceps (Figure-2) and hemostasis then performed on the edges of the donor area.

Graft preparation was done by removing any detrusor muscle fragments adhered to the mucosa. Dorsal and/or dorsolateral fixation of the graft to the corpora cavernosa was performed using a 5-0 PDS suture (Figure-2). The graft/urethra anastomosis was completed using 5-0 PDS (Figure-2). A 16F urethral catheter was inserted at the end the procedure. In patients with prior urinary diversion an 18F suprapubic catheter was used. A closed suction perineal drain was routinely inserted (Figure-2), the perineum was closed in layers, and a compressive dressing applied.

#### Postoperative Evaluation

Patients were hospitalized for 48 hours and then discharged with both urinary catheters in place. The urethral Foley catheter was removed in 21 days. A week later, the patient was submitted to uroflowmetry. If the maximum flow was equal to or greater than 14mL/s, the suprapubic urinary diversion was removed. At the intervals of 30-, 90- and 180-days post-operatory, repeated uroflowmetry was performed. After the time of the final evaluation, the PROMS questionnaire translated into Portuguese was reapplied.

Therapeutic success was defined as not requiring further procedures, Qmax above 12mL/s, post-void residual below 50mL, and the ability to insert a 15F flexible cystoscope if undergoing diagnostic cystoscopy.

### Statistical Analysis

Summary statistics are presented as percentages or mean ± SD, as appropriated.

## RESULTS

Patient characteristics are listed in Table-1. Mean age of patients was 46.14 years (± 11.231). Of 7 patients who underwent urethroplasties, 2 cases (28.5%) had penile urethral stricture and 5 cases (71.5%) showed bulbar urethral stenosis. The average length of the stricture was 50mm (35-60). Regarding the etiology of the stenosis, 3 cases (42.9%) were idiopathic, 3 cases (42.9%) were traumatic and 1 case (14.3) was iatrogenic causes. Two patients (28.6%) had previously undergone urethroplasty with a ventral oral mucosal graft and 3 patients (42.8%) were using suprapubic urinary diversion.

**Table 1:** Patient Characteristics.

Variables		Results
Number of patients		7
Age, mean ± standard deviation		46.14 ± 11.231
SAH, n (%)		2 (28.6)
Morbid obesity, n (%)		1 (14.3)
Presence of supra pubic shunt, n (%)		3 (42.8)
**Stenosis location, n (%)**		
	Penis	2 (28.5)
	Bulbar	5 (71.5)
	Pan anterior stenosis	0
**Etiology, n (%)**		
	Idiopathic	3 (42.9)
	Trauma	3 (42.9)
	Iatrogenic	1 (14.3)
Stenosis length (mm), mean ± standard deviation		50 (35-60)
**Previous urethroplasty, n (%)**		
	Yes	2 (28.6)
	No	5 (71.4)
Graft size (mm), mean ± standard deviation		52.86 ± 13.801
Time for graft extraction (min), mean ± standard deviation		46.43 ± 14.639
**Perioperative complications, n (%)**		
	No	5 (71.4)
	Insufficient graft	1 (14.3)
	Burned graft	1 (14.3)
**Postoperative complications, n (%)**		
	No	5 (71.4)
	Hematuria	2 (28.6)

The mean length of the harvested bladder mucosa graft was 52.86mm (± 13.801) and the time spent for its removal was 46.43min (± 14.64). Five patients (71.4%) had no perioperative complications and/or technical difficulties and went on to have their surgeries completed. However, in 2 patients (28.6%) problems occurred related to the graft extraction. In the first case, the bladder mucosa was burned by the laser, and, in the second situation, the graft was insufficient to complete the procedure. In both cases, the procedure was completed using oral mucosa and these patients were then excluded from the study. Two patients (28.6%) had minor hematuria between the twelfth and eighteenth postoperative day, although both did not need hospitalization and/or additional procedures.

All patients achieved the normalization of uroflowmetry (mL/s), sustained throughout the follow-up which occurred at post-operative intervals of 30-days (17.8 ± 3.271), 90-days (20.6 ± 5.413) and 180-days (19.6 ± 8.019) (Table-2).

**Table 2:** Comparison of pre and postoperative uroflowmetry (ml/s).

Variables	Results
Preoperative uroflowmetry (ml), mean ± standard deviation	4.0 ± 1.000
Postoperative uroflowmetry 30 days (ml), mean ± standard deviation	17.8 ± 3.271
Postoperative uroflowmetry 90 days (ml), mean ± standard deviation	20.6 ± 5.413
Postoperative uroflowmetry 180 (ml), mean ± standard deviation	19.6 ± 8.019

Table-3 summarizes the results of the PROMs translated into Portuguese applied before surgery and 6 months after.

**Table 3:** Comparison of USS PROMs parameters.

	Preoperative				Postoperative			
	n	Mean	Median	Standart	n	Mena	Median	Standart
LUTS Score	3	19.33	20.0	2.082	5	5.40	5.0	4.037
ICIQ-MLUTS Score	5	3.80	4.0	0.447	5	2.00	2.0	1.000
Peeling Picture Score	3	4.00	4.0	0.000	5	2.20	2.0	0.837
EQ-5D	5	0.6371	0.6896	0.1230	5	0.7285	0.6896	0.0532
EQ-VAS	5	58.00	50.0	25.884	5	84.00	90.0	20.736

There was an important improvement in the LUTS Score, with average scores dropping from 19.3 to 5.4. Similar improvements were also seen in the ICIQ-MLUTS Score (mean drop from 3.8 to 2.0) and Peeling Picture Score (mean drop from 4.0 to 2.2). In the variables related to an improvement in the quality of life, there was a mean increase from 0.6371 to 0.7285 in the EQ-5D and from 58.0 to 84.0 in the EQ-VAS, indicating a definite improvement in the quality-of-life following urethroplasty.

## DISCUSSION

The use of bladder mucosa for the treatment of complex urethral strictures was described for the first time by Memmelaar et al. in 1947 (7) for the treatment of 4 patients with hypospadias. Applying the knowledge and technology of that time, the grafts were harvested using an open technique and tubularized for the repair of hypospadias in a 1-stage procedure, obtaining patency in 3 out of 4 patients after 1 year.

Between 1980 to 1990, with the evolution of surgical techniques, anesthetics, and the use of antibiotics, several authors published their experiences using bladder mucosa as a graft. These grafts were applied principally for the treatment of hypospadias and other genitourinary malformations such as epispadias, always using the open technique for the mucosa removal.

Coleman et al. (19) used bladder mucosa as a tubular graft for the surgical repair of 9 hypospadias patients and obtained high complication rates whereby 6 patients incurred meatus stenoses and 1 incurred anastomosis stenosis.

Hendren et al. (20) operated on 35 patients with multi-operated hypospadias, epispadias and urethral strictures of traumatic origin while also using tubular grafts from bladder mucosa. Six meatal redundancies/eversions occurred, thereby requiring repair procedures.

In 47 patients with complicated hypospadias, Ransley et al. (21) performed urethral reconstruction using a combined graft of tubularized bladder mucosa associated with the foreskin for meatus reconstruction. Their aim was to avoid columnar metaplasia caused by the exposure of the bladder mucosa to dry environments. This exposure ends up producing meatal viscosity and occlusion. Of the 47 patients, meatal problems occurred in 18 patients (38%), fistulas occurred in 10 patients (21%) and anastomosis stenosis occurred in 1 patient (2%).

In the study by Decter et al. (22), 13 children were submitted to the correction of complicated hypospadias using tubularized vesical mucosal grafts. Their outcomes were that 5 patients (25%) developed meatal problems, 3 patients (18%) incurred restenosis and 2 patients (13%) incurred fistulas in a short-term follow-up.

In the study published by Mollard et al. (23), 76 patients were submitted to surgical cure of primary and multi-operated hypospadias using a tubularized bladder mucosa graft. Their outcomes revealed 18 cases (24%) of meatal stenoses/meatal redundancies, 10 patients (15%) incurred fistulas in the proximal anastomosis, and 3 patients (4.4%) incurred complete graft failure.

Keating et al. (8) described their experience with bladder mucosa whereby the harvesting of the graft used the open technique for repair in 11 patients between the ages of 10 and 30 years old. These patients incurred severe complications with prior hypospadias corrections. Outcomes revealed 2 patients incurring poor graft attachment resulting in the continuation of the penoscrotal hypospadias therefore requiring new procedures, 2 patients required dilations and urethrotomies between 8 and 9 weeks after the procedure, which, in the end, obtained a satisfactory result, and 1 patient incurred meatus eversion requiring self-catheterization. Satisfactory results that did not require new procedures were obtained in 7 patients. According to the author, the bladder mucosa behaves much in the same way as a bladder with exstrophy when in contact with the external environment. This may explain the major complications inherent to the procedure.

Monfort et al. (24) expanded the indication for the use of bladder mucosa for stenosis treatment for the bulbar urethra/membranous bulb, in 8 children excluding post hypospadias stenoses. The open technique was used to remove the vesical mucosal graft. In a mean follow-up of 2.3 years, the outcomes revealed that 2 children had a recurrence of stenosis where in the 1st patient the stenosis occurred in a site different from the grafting site. In the 2nd case, stenosis occurred in the graft site which had been previously tubularized.

Long-term results of 95 patients were published by Kinkead et al. (9). Their study was based on patients between the ages of 1 year - 21 years old. These patients underwent urethral reconstruction with autologous vesical mucosa graft for the repair of primary hypospadias, recurrent hypospadias and complex epispadias exstrophy cases. The authors performed a complete replacement of the urethra, an onlay patch or a combined repair with tubularized bladder mucosa associated with skin grafts or flaps. Published results were disappointing showing a high rate of reapproach (average of 2.7 procedures, ranging from 1 to 9 procedures, the main complication being meatal problems, fistulas, and restenosis.

Ösgök et al. (10) submitted 14 patients, with penoscrotal or scrotal hypospadias, to urethral repair using vesical mucosa grafts that were harvested using an open technique. The authors used a tubularized graft with only bladder mucosa or associated it with a foreskin graft and/or a tunica vaginalis graft. A 23-month follow-up revealed high rates of complications related to the meatus and to fistula formation.

Up to this point in time, most studies using free grafts from the bladder mucosa to correct anterior urethral diseases used tubularized grafts placed over a foley catheter, something which today we know is not recommended.

Morey and McAninch (6), Barbagli et al. (17) and Kulkarni et al. (18), described and popularized the use of grafts such as ventral, dorsal, and lateral dorsum onlay patches. From this point in time and leading forward, these became the main grafting techniques used regardless of the type of graft.

A retrospective study published by Fu et al. (25) reported 10 years of experience in the surgical cure of hypospadias in 294 patients. They used free grafts of bladder mucosa associated with the skin of the penile shaft using a ventral onlay patch, and then opted for making a wide coronal urethral meatus. A follow-up of up to 5 years revealed that fistula formation occurred in 27 patients (9.1%) and neourethral stenosis developed in 9 patients (3.1%) during this period.

A comparative study between bladder and oral mucosal grafts had been carried out by Marzorati et al. (26) in 11 patients with anterior urethral stenosis due to hypospadias or traumatic urethral stenosis, all multi-operated cases. Dorsal onlay free grafts were used, obtaining similar success rates (75%) when comparing the two groups in a mean follow-up of 50 months. Restenosis was found in 2 patients (25%), one with both types of graft.

Up to this point in time, every publication described the harvesting of the bladder mucosa using an open technique, which now, in the era of using minimally invasive procedures, has helped this type of graft to fall into disuse. Another factor that contributed to the decrease in the use of bladder mucosa were the superior results being obtained when using oral mucosa where the donor site allows for easy harvesting.

Wang Z et al. (11), however, using an important technical innovation for the collection of bladder mucosa, published their interesting study results. The innovation consisted of the endoscopic dissection of the vesical submucosa using a jet of water applied through equipment called a hybrid knife system. For the first stage of the hypospadias repair in 2 patients, the resultant graft was harvested and then implanted for the correction of a stenosis of the bulbar urethra in the 1st patient and the repair of a hypospadias relapsed in the 2nd. No complications occurred. The stage of harvesting of the graft took 35 and 30 minutes, respectively. A short follow-up of 4 months revealed an outcome where no recurrence of the disease developed in the 1st case, and that there was a good adhesion of the reconstructed urethral plate in the 2nd case.

Unlike the afore mentioned hybrid knife system, the Holmium laser (Ho-YAG) is an equipment that is easily available and widely used in urological procedures.

The first publication on the use of the Ho-YAG for the removal of bladder mucosa with the treatment of urethral stenosis was described by Figueiredo et al (16) in 2022. This study related the 18-month follow-up of a patient who presented a 6 cm idiopathic bulbar urethral stricture and resulted in a surgical success in absence of any perioperative or postoperative complications.

It was demonstrated, in our small series study, the possibility of harvesting a graft from bladder mucosa in a safe manner without any type of perioperative complications. Although hematuria did occur during the postoperative period in 2 patients, these were minor episodes, both without the need for hospitalization and/or additional procedures.

The mean harvesting time was relatively long (46.43 ± 14.64 minutes), which can be explained by the learning curve the technique required. Furthermore, 2 patients were excluded from our study due to problems related to the harvesting. In the first case, the dissection plane with the laser was very superficial, causing burns and retraction of the bladder mucosa. In the second case, the graft collected was insufficient in size to correct the stenosis. Both cases demonstrate the importance of adequate training so that harvesting is performed on the correct dissection plane. As already evidenced by previous authors (24), considering that the bladder is a dynamic organ, the exact measurement of the graft collected can be challenging for the urologist.

Exclusion criteria of patients included those presenting genitourinary malformations, a history of pelvic radiotherapy, a history of bladder cancer, and those having an indication for staged urethroplasty. It was decided to exclude genitourinary malformations because, in the past, the vast majority of studies used the bladder mucosa for the correction of multi-operated hypospadias, which has been shown to be a challenging surgery for any graft type.

According to past understanding, bladder mucosa does not behave well in dry environments, suffering columnar metaplasia that ends up producing increased viscosity and redundancy21. It was therefore decided to exclude patients with indication for staged urethroplasty and also those with the stricture of the glans urethra, in order to prevent meatal problems.

Bladder mucosal grafts are tenacious, soft, and tear easily. Due to these qualities, it was decided to perform urethroplasty techniques that use the corpora cavernosa as a bed to avoid the formation of diverticula, and to facilitate the fixation of the graft within its recipient bed.

Despite this study showing limitations inherent to the low number of patients and having a short follow-up, we could observe a consistent improvement in the uroflowmetric parameters and also in all domains of the PROM questionnaire applied both preoperatively and 6 months post-operatively.

## CONCLUSIONS

Transurethral harvesting of bladder mucosa using the Holmium laser (Ho-YAG) is feasible and reproducible. Training is needed to avoid damaging the graft and obtain an appropriately sized graft. Our preliminary experience suggests that bladder mucosa grafts achieve comparable results to other grafts when used for dorsal onlay urethroplasty.

Further research is needed to confirm these results.
